# Impact of parent–adolescent bonding on school bullying and mental health in Vietnamese cultural setting: evidence from the global school-based health survey

**DOI:** 10.1186/s40359-019-0294-z

**Published:** 2019-03-18

**Authors:** Hoang Thuy Linh Nguyen, Keiko Nakamura, Kaoruko Seino, Saber Al-Sobaihi

**Affiliations:** 1 Department of Global Health Entrepreneurship, Division of Public Health, Graduate School of Tokyo Medical and Dental University, Tokyo, 113-8519 Japan; 2 Faculty of Public Health, Hue University of Medicine and Pharmacy, Hue University, Hue, Vietnam; 3 Promotion Committee for Healthy Cities, Tokyo, Japan; 4 WHO Collaborating Centre for Healthy Cities and Urban Policy Research, Tokyo, Japan

**Keywords:** Parent–adolescent bonding, School bullying, Mental health problems, Adolescent, Vietnam

## Abstract

**Background:**

The mental well-being of adolescents is a crucial issue affecting lives of both adults and young people. Bullying and mental health problems are important factors that can have a negative impact on the mental well-being of adolescents. Public awareness of mental health problems among adolescents is rapidly growing in Vietnam. However, current approaches to identifying risk factors influencing mental health problems do not pay attention to potentially protective factors. This study was performed to examine the associations between parent–adolescent bonding and mental health outcomes as protective elements during the adolescent period.

**Methods:**

Data collected from 3331 respondents in grade 8–12 as part of the Vietnam Global School-based Student Health Survey (GSHS) 2013 was used for the analysis. A three-stage cluster sample design was used to produce data representative of students. Multivariate logistic regression analysis was performed to examine the association of demographic characteristics and data regarding parent–adolescent bonding associations with status of mental health problems in adolescents.

**Results:**

Parental understanding, parental monitoring were significantly associated with reduced likelihood of being bullied and mental health problems (*P <* 0.05). However, parental control was significantly associated with greater likelihoods of being physically attacked (adjusted odd ratio (aOR) = 1.36, 95%CI, 1.06, 1.75) and mental health problems, such as suicidal ideation, and loneliness (aOR = 1.96, 95%CI, 1.49, 2.57, aOR = 2.35, 95%CI, 1.75, 3.15, respectively), after adjusting for potential confounders.

**Conclusions:**

The study indicated the significant associations between parental understanding, monitoring and control in a proxy of parent–adolescent bonding and mental well-being during the period of adolescent rebellion. Thus, parent–adolescent bonding in Southeast Asian cultural context may provide an effective means to promote the mental well-being of adolescents.

## Background

Mental well-being is a fundamental component of the World Health Organization (WHO) definition of health, and the core of mental health action with the principle “no health without mental health” was globally accepted [1]. The mental well-being of adolescents is a crucial issue affecting lives of both adults and young people. Suicide is among the top causes of death among teenagers [2], and the prevalence rates of ever suicidal ideation and attempted suicide are high among Asian youths [3] with estimated values of 11.7 and 2.4%, respectively, in six ASEAN member states [3, 4]. Bullying and mental health problems are important phenomena that can have a negative impact on mental well-being among adolescents.

There is a growing awareness regarding mental health problems in Vietnam [5, 6]. The Survey Assessment of Vietnamese Youth (SAVY I, SAVY II) showed that more than 30% of adolescents self-reported lifestyle experiences of low mood, and the prevalence rates of suicide behaviors were 5.28% (SAVY I) and 12.21% (SAVY II) [7]. Poor mental health (anxiety, depression and suicidal ideation) was shown to be common among adolescents in a number of provinces in Vietnam [8], and a number of risk factors were shown to be associated with mental health problems, including female sex, belonging to an ethic minority, illiteracy, exposed to violence, stress related to education loans, following a religion other than Buddhism, and living in a wealthier family [7].

Bullying behaviors is a widespread phenomenon in childhood and adolescence. The core issues of violence among adolescents in school magnify concerns about school bullying, which is not only significant public health issue but is also a possible determinant of poor mental well-being among adolescents [9]. Moreover, studies performed in different countries have indicated that bullying at school is associated with psychological distress and suicidal behavior in adolescents [10–13].

The family environment is an important factor related to mental health problems and school bullying. Parent–child relations are thought to be important contributors to children’s growth, and personality development as well as having important effects on children’s health and mental well-being [14, 15]. Permissive parents tend to indulge their children, encourage them to be autonomous in decision making, and rarely punish them for misbehavior. In contrast, authoritarian parents are strict, controlling, and consistently punish children for disobedience. Authoritative parents take an intermediate approach, aiming to have open communication with their children to understand the consequences of their behaviors and decisions. A concept of a ‘parent-adolescent bonding’ is considered to be consisted of two dimensions, parental care and parental overprotection [16]; the latter is characterized by excessive contact and interference to independent behaviors [17, 18]. Several studies performed in Western countries and in Asia have indicated that not only perceived parenting, but also parenting styles as well as parental care, over-control, conflict, and warmth are associated with adolescent mental health problems [19]. Studies in the USA conducted among adolescents aged 11–18 years old indicated that children with positive feelings toward their parents tended to have good mental health, while those with negative feelings were more likely to exhibit problem behavior [20]. In traditional Vietnamese culture, the multigenerational interaction plays a more important role in individuals’ lives, parenting styles draw mostly on Buddhist beliefs, emphasizing the interdependence of the family, respect for adults, and obedience toward parents [21]. Vietnam, one of top three countries with high GDP growth rates among Southeast Asia [22], is experiencing rapid socio-economical changes in society, such as increased internal migration from rural to urban areas, changes in family structure, and changes in roles of parents in modernized families [21]. How does the picture describe the role of parent–child bonding and mental well-being during the adolescent period in specific context? There has been limited research regarding this topic in Vietnam.

The present study was performed to identify if parental understanding, parental monitoring, and parental control in the proxy of parent–adolescent bonding are associated with (1) school bullying including being bullied, being physical attacked and (2) mental health problems (loneliness and suicide ideation) after adjusting for potential confounding factors.

## Methods

### Data source

The present study was performed using the publicly available data obtained as part of the Vietnam Global School-based Student Health Survey (GSHS) in 2013, which is available online [23]. The 2013 Vietnam GSHS used a three-stage cluster sampling design to recruit a nationally representative sample of school students in grades 8–12 in Vietnam. Provinces in the first stage, and schools in the second stage were selected with probability proportional to enrollment size. At the third stage, all students in randomly selected classes were eligible for inclusion in the study. The total sample consisted of 3331 students aged 12–17 years old. Students self-reported their responses to each question in the GSHS questionnaire and the overall response rate was 96%.

### Survey instruments

The questionnaires developed by the WHO and Centers for Disease Control and Prevention (CDC) for use in the GSHS were used to collect information from school students in grades 8–12. In some previous validation studies, GSHS was reported to have acceptable validity [24].

### Study variables

Four binary outcome variables were measured: (1) having been bullied (2) having been physically attacked (3) loneliness and (4) suicidal ideation.

School bullying was defined as aggressive behavior by a student or group of student with a power imbalance and potential to be repeated. (1) Having been bullied was identified with the question “How many days were you bullied during the past 30 days?” and the response was recoded as “yes” for answer of one or more days or “no” (2) Having been physically attacked was examined with the question “How many times were you physically attacked during the past 12 months?” and was recorded by “yes” for answer of one or more times or “no”.

Mental health problems among adolescents were defined as feelings of loneliness and suicidal ideation. (3) The incidence of loneliness was examined using the question “How often have you felt lonely during the past 12 months?” with responses ranging from “never” to “always.” The responses were dichotomized to “lonely” meaning most of the time/always or “no” meaning “never/rarely or sometimes.” (4) Suicidal ideation was examined with the question “Did you ever seriously consider attempting suicide during the past 12 months?” with a binary response of “yes” or “no.”

The analysis included a number of independent variables that may influence the likelihood of school bullying and mental well-being of adolescents: gender (male, female), education level (junior high school, senior high school), and food insecurity (never/rarely, sometimes, most of the time/always).

Parental understanding, parental monitoring and parental control are components of a proxy of parent–adolescent bonding [16]. Parental understanding and parental monitoring were identified via separate questions, i.e., “How often did your parents or guardians understand your problems and worries during the past 30 days?” and “How often did your parents or guardians really know what you were doing with your free time during the past 30 days?” Parental control was examined with the question “How often did your parents or guardians go through your things without your approval during the past 30 days?” The responses to these questions were “never,” “rarely,” “sometimes,” “most of the time,” and “always.” These variables were recoded and classified as “yes,” which included “most of the time/always,” and “no,” which included “never,” “rarely,” and “sometimes.”

Relationships with friends were defined as having close friends and the respondents reported that the majority of friends were mostly supportive always or most of the time.

### Data analysis

In descriptive analysis, categorical variables were summarized using proportions and were then presented in tables and with significance of differences determined by Pearson Chi Square test for categorical variables. All cases which have missing values of selected variables in the public dataset were excluded, and 2968 subjects were finally included in the analysis.

Bivariate analysis was then performed to test for associations between the outcome variables. i.e., school bullying, and mental health problems, and other independent variables.

A multivariate logistic regression model was used to evaluate associations between outcome variables and risk factors related to parental understanding, parent monitoring and parental control after adjustment for potential independent variables (gender, education levels, food insecurity, and relationship with friends). The Hormer and Lemeshow Goodness-of-Fit Test with *P* > 0.05 was used to assess the goodness of fit model. In all analyses, *P* <  0.05 was taken to indicate statistical significance.

The data were analyzed using SPSS version 23.0 (SPSS Inc., Chicago, IL).

## Results

A total of 3331 subjects completed the self-reported questionnaire used in this survey. Overall, 46.9% (*n* = 1765) of the 3331 adolescents were boys, and there were no significant differences in gender distribution according to education level. Only 34 of the adolescents (1.0%) reported food insecurity “most of the time/always,” and there were no significant differences in rate of food insecurity between junior high school and senior high school (*P >* 0.05). The majority of the respondents reported having close friends (93.0%) that were supportive always/most of the time (51.6%). The percentages of respondents that reported parental understanding of their problems and parental monitoring of their free time activities were 31.3 and 38.5%, respectively. Parental understanding and monitoring showed significant differences according to education level (*P* <  0.05). Moreover, the percentage of respondents reporting parental control was 14.2% (465 of the total of 3331 adolescents), and the value did not differ significantly according to education level (*P* > 0.05) (Table 1).

**Table 1:** Descriptive statistics by education level among Vietnamese adolescents

	Total	Junior high school		Senior high school		P*
	*n* = 3331	n	%	n	%	
Sex						
Male	1557	731	46.3	655	47.2	0.615
Female	1765	849	53.7	733	52.8	
*Missing value*	9					
Food insecurity						
Never/Rarely	2593	1265	80.1	1073	77.3	0.173
Sometimes	689	302	19.1	300	21.6	
Most of the time/Always	34	13	0.8	15	1.1	
*Missing value*	*15*					
Parental understanding ‘Parents understood problems’						
Yes	1038	532	33.7	390	28.1	0.001
No	2283	1048	66.3	998	71.9	
*Missing value*	*10*					
Parental monitoring ‘Parents were aware of free time activities’						
Yes	1274	654	41.4	492	35.4	0.001
No	2035	926	58.6	896	64.6	
*Missing value*	*22*					
Parental control ‘Parents went through things without permission’						
Yes	465	230	14.6	188	13.5	0.429
No	2814	1350	85.4	1200	86.5	
*Missing value*	*52*					
Supportive friends						
Yes	1669	797	50.4	716	51.6	0.535
No	1644	783	49.6	48	48.4	
*Missing value*	*18*					
Close friendships						
0	173	53	3.4	97	7.0	< 0.001
≥1	3138	1527	96.6	1291	93.0	
*Missing value*	*20*					
Having been physically attacked						
One or more times	734	433	27.4	197	14.2	< 0.001
No	2582	1147	72.6	1191	85.8	
*Missing value*	*15*					
Having been in physical fight						
One or more times	576	325	20.6	166	12.0	< 0.001
No	2731	1255	79.4	1222	88.0	
*Missing value*	*24*					
Having been bullied						
One or more days	744	420	26.6	255	18.4	< 0.001
No	2456	1160	73.4	1133	81.6	
*Missing value*	*131*					
Mental health						
Loneliness						
Yes	367	137	8.7	178	12.8	< 0.001
No	2910	1443	91.3	1210	87.2	
*Missing value*	*54*					
Suicidal ideation						
Yes	539	215	13.6	258	18.6	< 0.001
No	2750	1365	86.4	1130	81.4	
*Missing value*	*42*					

*Excluding all missing values *n* = 2968

Table 1 also shows the prevalence rates of being bullied, being physically attacked and mental health problems. The rates of being physically attacked or bullied in school for one or more days among the respondents were 23.3 and 22.1%, respectively. Among the study population, 11.2 and 16.4% reported feeling lonely and having thoughts of suicide, respectively, and mental health problems showed significant differences according to education level (*P* <  0.05*).*

Table 2 shows the results of logistic regression analysis regarding school bullying (being bullied or being physically attacked) with adolescents’ characteristic, parent–adolescent bonding, and support of friends. The factors included in the analysis included education level, parental understanding, parental monitoring, supportive friends and close friendships, and showed significant relations with likelihood of being bullied *(P* <  0.05*).* After adjusting the confounding variables, adolescents with parental monitoring their children’s free time activities had 0.78 times lower rates of being bullied compared to those without parental monitoring (adjusted odd ratio (aOR) =0.78, 95%CI, 0.63, 0.95). Moreover, education level, and supportive friends in school remained significantly associated with rate of being bullied (aOR =1.69, 95%CI, 1.41, 2.03, aOR = 0.64, 95%CI, 0.53, 0.76, respectively) on multivariate logistic regression analysis.

**Table 2:** Associations between parent–adolescent bonding and bullying/victimization among Vietnamese adolescents

	Having been bullied		Having been physically attacked	
	OR (95%CI)	aOR (95%CI)	OR (95%CI)	aOR (95%CI)
Sex				
Male	0.92 (0.77, 1.09)	0.92 (0.77, 1.10)	1.85 (1.55, 2.21)***	1.92 (1.60, 2.31) ***
Female	1	1	1	1
Education level				
Junior high school	1.61 (1.35, 1.92)***	1.69 (1.41, 2.03)***	2.28 (1.89, 2.75)***	2.46 (2.03, 2.98)***
Senior high school	1	1	1	1
Food insecurity				
Never/Rarely	0.65 (0.29, 1.49)	0.70 (0.30, 1.62)	0.51 (0.23, 1.14)	0.50 (0.22, 1.17)
Sometimes	1.10 (0.48, 2.54)	1.11 (0.47, 2.63)	0.78 (0.35, 1.77)	0.76 (0.32, 1.80)
Most of the time/Always	1	1	1	1
Parental understanding ‘Parents understood problems’				
Yes	0.73 (0.60, 0.89)***	0.90 (0.73, 1.11)	0.82 (0.67, 0.99)*	0.95 (0.76, 1.19)
No	1	1	1	1
Parental monitoring ‘Parents were aware of free time activities’				
Yes	0.69 (0.58, 0.83)***	0.78 (0.63, 0.95)*	0.65 (0.54, 0.78)***	0.67 (0.54, 0.82)***
No	1	1	1	1
Parental control ‘Parents went through things without permission’				
Yes	1.22 (0.96, 1.55)	1.26 (0.99, 1.61)	1.37 (1.08, 1.74)**	1.36 (1.06, 1.75)*
No	1	1	1	1
Supportive friends				
Yes	0.58 (0.49, 0.70)***	0.64 (0.53, 0.76)***	0.65 (0.54, 0.78)***	0.71 (0.59, 0.86)***
No	1	1	1	1
Close friendships				
Yes	0.69 (0.48, 0.99)*	0.74 (0.51, 1.07)	0.81 (0.55, 1.19)	0.86 (0.57, 1.28)
No	1	1	1	1

* *P* < 0.05, ** *P* < 0.01, ***P < 0.001

*OR* Odds ratio

aOR adjusted odds radio (adjusted for sex, education level, food insecurity, parent-adolescent bonding, supportive friends, close friendship)

*CI* confidence interval

As shown in Table 2, being physically attacked had significant associations with sex, education level, the proxy of parent-adolescent bonding, and supportive friends in school *(P* <  0.05*).* Multivariate analysis indicated that adolescents with parental monitoring were 0.67 times less likely to have been physically attacked than those without parental monitoring, while those with parental control were 1.36 times more likely to have been physically attacked than those without parental control (aOR =0.67, 95%CI, 0.54, 0.82, aOR =1.36, 95%CI, 1.06, 1.75, respectively).

Table 3 shows factors suggested to be associated with mental health problems among Vietnamese adolescents, which included loneliness and suicidal ideation. Bivariate analysis indicated significant associations of parental understanding, parental monitoring, and parental control in a proxy of parent-adolescent bonding with loneliness and suicidal ideation. Parent-adolescent bonding remained significant predictors of mental health status with the addition of potential confounding factors into the logistic regression model, such as sex, education level, food insecurity, supportive friends, close friends and some of variables related to bullying. In particular, adolescents with parental understanding, parental monitoring had significantly lower rates of suicidal ideation (aOR =0.61, 95%CI, 0.46, 0.81, aOR =0.52, 95%CI, 0.40, 0.67, respectively) and parental monitoring had significantly lower rates of loneliness (aOR =0.62, 95%CI, 0.46, 0.83) while the rates of suicidal ideation and loneliness were approximately double in adolescents with parental control (aOR =1.96, 95%CI, 1.49, 2.57, aOR =2.35, 95%CI, 1.75, 3.15, respectively).

**Table 3:** Association between parent-adolescent bonding and mental health among Vietnamese adolescents

	Suicidal ideation		Loneliness	
	OR (95%CI)	aOR (95%CI)	OR (95%CI)	aOR (95%CI)
Sex				
Male	0.51 (0.41, 0.62) ***	0.42 (0.34, 0.53)***	0.79 (0.63, 1.01)	0.71 (0.55, 0.92)*
Female	1	1	1	1
Education Level				
Junior high school	0.69 (0.57, 0.84) ***	0.67 (0.54, 0.83)***	0.65 (0.51, 0.82)***	0.63 (0.49, 0.81)***
Senior high school	1	1	1	1
Food insecurity				
Never/Rarely	0.62 (0.25, 1.54)	0.82 (0.31, 2.15)	0.49 (0.18, 1.30)	0.68 (0.24, 1.92)
Sometimes	1.00 (0.40, 2.52)	1.04 (0.39, 2.76)	0.76 (0.28, 2.04)	0.84 (0.29, 2.40)
Most of the time/Always	1	1	1	1
Parental understanding ‘Parents understood problems’				
Yes	0.41 (0.32, 0.53) ***	0.61 (0.46, 0.81)***	0.58 (0.44, 0.77)***	0.81 (0.58, 1.10)
No	1	1	1	1
Parental monitoring ‘Parents were aware of free time activities’				
Yes	0.39 (0.31, 0.50) ***	0.52 (0.40, 0.67)***	0.51 (0.39, 0.66)***	0.62 (0.46, 0.83)***
No	1	1	1	1
Parental control ‘Parents went through things without permission’				
Yes	1.75 (1.36, 2.25)***	1.96 (1.49, 2.57)***	2.29 (1.73, 3.02)***	2.35 (1.75, 3.15)***
No	1	1	1	1
Supportive friends				
Yes	0.66 (0.54, 0.80)***	0.84 (0.68, 1.04)	0.71 (0.56, 0.90)*	0.88 (0.68, 1.13)
No	1	1	1	1
Close friendships				
Yes	0.33 (0.23, 0.47)***	0.41 (0.28, 0.60)***	0.24 (0.17, 0.35)***	0.31 (0.21, 0.45)***
No	1	1	1	1
Having been physically attacked				
Yes	1.47 (1.18, 1.85) ***	1.06 (0.79, 1.41)	1.48 (1.14, 1.93)**	0.99 (0.71, 1.39)
No	1	1	1	1
Having been in physical fight				
Yes	1.84 (1.45, 2.33)***	2.02 (1.50, 2.72)***	1.72 (1.30, 2.27)***	1.53 (1.09, 2.15)*
No	1	1	1	1
Having been bullied				
Yes	1.72 (1.39, 2.14)***	1.29 (1.01, 1.66)*	2.11 (1.65, 2.71)***	1.80 (1.35, 2.38)***
No	1	1	1	1

* *P* < 0.05, ** *P* < 0.01, ***P < 0.001

*OR* Odds ratio

aOR adjusted odds radio (adjusted for sex, education level, food insecurity, parent-adolescent bonding, supportive friends, close friendships, having been physically attacked, having been in physical fight, having been bullied)

*CI* confidence interval

## Discussion

The results of the present study suggest that the parent–adolescent relationship was associated with mental health of adolescents. This study also demonstrated association between adolescent mental well-being and gender, education level, and relationships with friends. In the proxy of parent-adolescent bonding, parental understanding and parental monitoring were significantly associated with reducing the likelihoods of school bullying and mental health problems, while parental control was associated with increased rates of being bullied in school or have mental health problems among adolescents.

The results of this study indicated that school bullying and mental health problems are important concerns among school-going adolescents in Vietnam, and being bullied was related to higher likelihood of mental health problems, as reported in previous studies [4, 8, 9, 13, 25–28]. There were significant differences in rates of being bullied, being physically attacked, loneliness, and suicidal ideation according to education levels and gender in this study [26]. School bullying is more common in junior high school compared to senior high school [29]. This may be because senior students have undergone more physical and psychosocial development than their younger counterparts, and are therefore better able to protect themselves [30]. In contrast, younger students have less likelihood of mental health problems [4].

The school environment and family climate have important roles in promoting school health as it related to bullying [31]. Parental understanding and parental monitoring were shown here to be related to a lower likelihood of adolescent being bullied or being physically attacked. Parental interest in their children’s free time activities and problems has a protective effect against bullying in school from student’s point of view. Socioeconomic changes also affect parental care, as modern parents are often busy with work and sometimes do not know about their children’s problems during adolescent development. The result presented here indicated that a high level of parental concern has a positive association to their adolescent children.

The association between of parent–adolescent bonding and mental well-being observed in this study was consistent with previous research [19, 32, 33]. Although modern trends emphasize adolescents’ competence and needs for independence, parents may be certain that their involvement in the lives of their adolescent children promotes mental health [33]. Moreover, a strong respect for their elders is inculcated in children in cultural context of most Southeast Asian countries, including Vietnam. In particular, communication between parents and adolescents is based on respectful conversation about family roles, relationships, and other social issues. As noted above, Southeast Asian culture generally emphasizes respect for authority. Southeast Asian parents are more restrictive and more control-oriented than their European and American counterparts, and they tend to use more commands and attempt to directly control their children’s attention [34]. With regard to the findings of this study, it is therefore noteworthy that parental understanding and monitoring seem to play a protective role in improving mental well-being, while parental control is a risk factor for increasing mental health problems during the adolescent period. These results are consistent with previous findings indicating that a high level of parental involvement is related to reduce likelihood of poor mental health among adolescents [19, 35], while a lack of parental warmth and high maternal over-control are associated with a wide range of psychological problems, including depression, suicidal behavior, and self-harm among adolescents [36–38].

In this observational study, directions of relationship between parental-adolescent bonding and mental health among adolescence can be interpreted in both ways [39]. One is a pathway from the characteristics of parental bonding influence to adolescents’ mental health presented by a multivariable logistic regression model. However, the inference of the potential reverse association is still possible. An example of alternative causal inference is that antisocial behavior of children increases monitoring by parents. Future research is required to address the causal pathway with quasi-experimental designs [39].

Adolescence is a time characterized by significant rapid neurological, cognitive and social changes for the integration of new and diverse experiences in relation to the world and themselves. Adolescence also presents the dilemma of maintaining a connection with parents while exploring new social roles away from the family and developing relationships with peers [40]. Rapid economic growth together with a lack of social infrastructure support resulted in increased pressure on families, threatening their traditional ability to socialize children into adaptively functioning adults [41, 42], therefore, parental–adolescents bonding may play critical role in leading children in the next level of social functioning [40]. Public health initiatives encouraging parents to maintain a connection with their adolescents are considered to help alter the general impression, and even the context of adolescent disinterest and rebellion [40].

The present study was performed using data collected by the WHO and CDC with a standardized questionnaire and methods, with a nationally representative sample size, and an appropriate sampling method. However, the study had several limitations. First, the cross-sectional survey could not make causal inferences. Second, as with many earlier studies in this areas, the outcome variables of this study (having been bullied, having been physically attacked, loneliness, and suicidal ideation) also the predictor related to proxy of parent-adolescents bonding were assessed with a single item question instead of a multi- items scale such as: instrument used in public health for depression by The Center for Epidemiological Studies-Depression Scale (CES-D) with 20 self-reported items; or Parental Bonding Instrument (PBI) consisting of 25 items, which could reduce validity and reliability. Therefore, the further validation measurement of this topic in Vietnam context may be required. Third, mental health measurements were dependent on the variables used in the GSHS survey in Vietnam. Therefore, single item was used for evaluation of adolescent-parent bonding instead of multi-items scale, and several adolescent mental health issues (depression, insomnia, and self-harm) were not evaluated. Finally, the results may have been affected by recall bias, due to use of self-reported responses.

## Conclusions

The results of this study indicated that the parent–adolescent connection had a significant association with mental well-being during the adolescent period. Parental understanding and parental monitoring in a proxy of parent-adolescent bonding have associated factors increased mental well-being of young people, while parental control was a risk factors during the period of adolescent rebellion. These findings suggest that focusing of the parent–adolescent connection in Southeast Asian cultural context may provide an effective means to promote mental well-being among adolescents. Further, Vietnamese parents should also participate in psychological education programs to raise awareness of how certain types of interactions with young people may represent strategies for reducing mental health problems and promoting a healthy school environment.

## Abbreviations

CDC: Centers for Disease Control and Prevention
GSHS: Global School-based Student Health Survey
SAVY: Survey Assessment of Vietnamese Youth
WHO: World Health Organization
